# THE PRESENCE OF UNKNOWN POLIO ENGAGEMENT CONFIRMED BY ELECTROMYOGRAPHY AND MUSCLE TESTING

**DOI:** 10.2340/jrm.v57.40718

**Published:** 2025-01-08

**Authors:** Waleed AL-NAJJAR, David KRABBE, Joakim STRANDBERG, Katharina S. SUNNERHAGEN

**Affiliations:** 1 Borås Hospital, Region Västra Götaland, Borås, Sweden; 2 Section for Clinical Neuroscience, Institute of Neuroscience and Physiology, the Sahlgrenska Academy, University of Gothenburg, Gothenburg, Sweden; 3 Department of Rehabilitation Medicine, Sahlgrenska University Hospital, Gothenburg, Sweden; 4 Department of Clinical Neurophysiology, Sahlgrenska University Hospital, Gothenburg, Sweden

**Keywords:** late effects of polio, polio rehabilitation, effectiveness of EMG, unperceived weakness, polio

## Abstract

**Objective:**

To evaluate the usefulness of electromyography at a polio clinic in identifying unperceived muscle denervation. Second, to compare people who perceived themselves as weak in 1 or both legs with those who did not.

**Design:**

Cross-sectional study.

**Subjects:**

The study included 542 persons with late effects of polio in Sweden. Mean age 58 at the first visit, 312 were female. Data used are from a clinical quality registry.

**Methods:**

At the first visit patients answered sent-out questionnaires. They underwent an electromyography test, walk test, and muscle strength assessment.

**Results:**

Electromyography identified signs of chronic neurogenic changes in lower limb muscles (*n* = 260) where the patient reported no previous involvement, comprising 239 of the participants. Significant differences in values shows that the group who did not perceive themselves as weak exhibited better performance, demonstrating greater speed, strength, and reduced reliance on wheelchairs.

**Conclusion:**

Electromyography can identify signs of muscle denervation in association with weakness in people with late effects of polio who perceived themselves as healthy. This comparison suggests that those that do not perceive weakness may unknowingly overuse their muscles. These findings contribute to our understanding of the late effects of polio and importance of early detection and rehabilitation.

## LAY ABSTRACT

In this study, researchers investigated the usefulness of electromyography in identifying muscle denervation in individuals with late effects of polio. They included 542 people in Sweden who had experienced polio and underwent various tests including electromyography, walk tests, and muscle strength assessments. The results showed that EMG was effective in identifying chronic neurogenic changes associated with muscle weakness, even in individuals who did not perceive themselves as weak. Those who were unaware of their weakness tended to exhibit greater walking speed and strength, and were less likely to rely on wheelchairs compared with those who were aware of their weakness. This study demonstrates that electromyography testing can identify effects of prior polio engagement in muscles perceived as normal, and thus provide valuable insights into subclinical affected muscles and thereby help prevent overuse of those muscles.

About 12–20 million individuals worldwide are living with sequalae of polio long after the acute infection (1). In Sweden, the estimated prevalence is more than 5,000 people (2). The polio virus can affect the motor nervous system, leading to local or generalized muscle weakness and leaving many individuals with residual symptoms known as late effects of polio. Studies have reported that 50–85% of individuals diagnosed with polio experience new problems when presenting at the polio clinic (3). These problems may arise from the biomechanical alterations in walking and mobility due to chronic musculoskeletal deformities and weakness caused by underlying polio. People can also experience biomechanical pain involving joint, soft tissue, or neural structures. Muscle, bursa, and tendon overuse syndrome may also occur (4).

However, the presentation of the infection might be unforeseen and the occurrence of remaining symptoms is not always clear. In fact, the infection may not be severe enough to cause observable muscle weakness, such as asymmetric paresis (5). Polio involvement can manifest as having had polio in 1 extremity without any other symptoms, and it may not be clear to the person who survived the acute episode that there has been more involvement in another part of the body. The stability period, defined as the period after the acute phase and recovery, can be seen as a new life with remaining impairments (late polio). During this stable period, people with polio who are unaware of their condition tend to compensate functionally, relying on muscle groups with good muscle function due to compensatory reinnervation and enlarged muscle fibres. These people may lack a reference to compare with, such as how their legs should function normally. Unawareness of these subtle changes can lead people with late effects of polio to overuse their muscles and to delay seeking clinical assistance (6).

Upon motor fibre degeneration caused by acute polio infection, the denervated muscle fibres can be reinnervated by axonal sprouting from remaining motor fibres, increasing the size of remaining motor units. If this process is effective, reinnervation might more or less totally compensate for slowly ongoing denervation, creating a dynamic balance and stable muscle function. Eventually, the motor neurons may no longer be able to maintain the axonal sprouts (4), leading to an imbalance with more denervation than reinnervation. The resulting decline will lead to a rapid loss of muscle function, which cannot be explained as part of the normal ageing process (7), and many previously subclinically affected limbs with polio will shift to a clinical phase with new onset weakness (8). These changes can be revealed by electromyography (EMG) and muscle strength testing, providing an explanation for the new symptom of weakness.

EMG is a diagnostic technique used to evaluate the electrophysiology of motor units, providing evidence of ongoing and/or previously undergone denervation (9). It should be part of the polio work-up to identifying the extent of motor unit deterioration (10). EMG can also provide additional information about the frequency and extent of reinnervation in clinically unaffected muscles of people with polio (11). The extension of motor neuron impairment in unaware people with impaired lower limb function complicates the rehabilitation process.

However, it is unclear whether the unawareness of the extension of the polio infection is true only for individual cases or whether this can be observed in a cohort of polio survivors and, if so, how frequent this is. This knowledge gap needs to be explored. The aim of the present study was to assess whether EMG can identify signs of muscle denervation in association with muscle weakness in people with late effects of polio, particularly those with uncertain knowledge of the extent of the infection. The secondary aim was to compare walking ability, muscle strength index, and the use of mobility aids between those aware and those unaware of their weakness at follow-up. Early detection of the subclinical phase would enable the rehabilitation team to develop new plans and assist people to modify their daily routines according to their abilities, potentially improving their quality of life.

## METHODS

### Study design and population

This is a registry based cross-sectional study. Data from community-dwelling people listed at a polio clinic were retrieved from a clinical quality registry (covering a population of around 2,000,000 inhabitants, which was 1/5 of the population of Sweden).

Data from 842 people diagnosed with polio and living with late effects of polio were available in this study. All people were listed at the polio clinic at Sahlgrenska University Hospital, Gothenburg, Sweden (12), and resided mainly in the western region of Sweden. People visited the clinic either by self-referral or through referral from another clinic. Prior to their first visit, they were sent a questionnaire to gather information on their history, weakness, and difficulties experienced during the acute phase of polio, as well as the present situation, including possible comorbidities. The first visit occurred during 1994–2015. The inclusion criteria were: (*i*) the person had an EMG performed on lower limbs prior to or at the first visit – the muscles addressed were quadriceps, hamstrings, tibialis anterior, and gastrocnemius, muscles needed to stabilize joints for gait; (*ii*) the person answered and returned the postal questionnaire.

### Procedure

During their first visit to the clinic, the following data were collected: muscle strength (and possible atrophy), use of mobility aids, gait speed, personal characteristics (age and sex), polio classification, country of birth. Those who had not undergone an EMG prior to their visit were then referred for EMG testing, which was performed at the clinical neurophysiology department at Sahlgrenska University Hospital. The polio clinic is organized as a multidisciplinary team, including physicians, occupational therapists, nurses, secretary, and social workers. All people underwent basic chemical laboratory examinations and were examined by the team during their first visit (13).

### Variables

Gait performance was evaluated by using a 30-m walk test (WT) at both comfortable and maximum speed (14). Trained physiotherapists at the polio clinic conducted the assessment. In the present study, the maximum speed values were used. Age and sex normative values from a healthy community-living population (at least 10 men and 10 women for each decade) were available for these tests, and the results are presented in percentages (15).

A standardized test of muscle strength (isometric and isokinetic test) was performed using the Biodex® multi-joint system 3 PRO dynamometers (https://biodexrehab.com/our-products/). The patients were seated comfortably with their back against a back rest. A seatbelt was strapped around the shoulders, waist, and thigh to avoid unexpected movements. Isokinetic muscle strength for concentric knee extensors and knee flexors was measured at a velocity of 60°/s for both sides. This velocity can represent pedalling of a bicycle and the average velocity of the knee joint when walking. The peak isometric strength of foot dorsal flexor and plantar flexors at a 30° ankle angle of knee flexion and 0° ankle angle was also measured. For this study, muscle strength measurements were transformed into a muscle index representing all 8 measurements, allowing all muscle groups to be weighted equally (7). The muscle strength index was calculated by constructing a factor for each muscle group, with strongest muscle group (knee extension, 60°/s) given a factor of 1. The factors for the other muscle group corresponded to the ratio in the healthy subjects between their average muscle strength and the average knee extensor muscle strength (Newton metres). Accordingly, the index was calculated as follows: 1×knee extension strength (right and left) + 2.2×knee flexion strength (right and left) +1.5×foot plantar flexion strength (right and left) +4.5×foot dorsal flexion strength (right and left) (7). The demand to have tested 8 different muscle groups in the limbs leads to missing subjects as not all could move the limb against gravity. In this study, people with late effects of polio were classified into 2 groups: an aware group with symptoms of weakness and an unaware group reporting no symptoms from an extremity where EMG changes were noted.

### Statistical analysis

The study sample was analysed with descriptive statistics (mean, min/max, SD). Independent sample *t*-test and Pearson’s χ^2^ tests were performed to detect possible differences between the aware and unaware groups. The comparison between the 2 groups was conducted for the 30-m walk test, muscle strength, and use of mobility aids. The values of the 30-m walk test were adjusted for sex and age and expressed as percentages based on reference values of a random population aged 40–80 (15). The remaining older ages were normalized to the closest reference to their age and sex. Use of mobility aids by people with late effects of polio was divided into 2 categories: the first included people walking with or without mobility aids and the second category were mobile using manual or electric wheelchairs. The proportion of missing values varied among the variables. To retain as many observations as possible, all available data were used.

Data analysis was performed using IBM SPSS version 26 (released 2019. IBM SPSS Statistics for Windows, Version 26.0; IBM Corp, Armonk, NY, USA). All statistical tests were two-tailed, at an alpha level of 5%.

### Ethical considerations

The study was approved by the Swedish Ethical Review Authority, Dnr 2022-02297-01. Data files were pseudonymized. Swedish quality registers are subject to the Personal Data Act (Swedish law No. SFS 1998:204), which allows data collection for quality control and without informed consent from the patient with an opt-out possibility. Research on quality registry data require ethical approval.

### Data availability statement

Due to regulations in Sweden, the data cannot be made publicly available. Researchers may apply to Professor Katharina Stibrant Sunnerhagen to access anonymized data after obtaining the necessary approvals.

## RESULTS

**Fig. 1:**
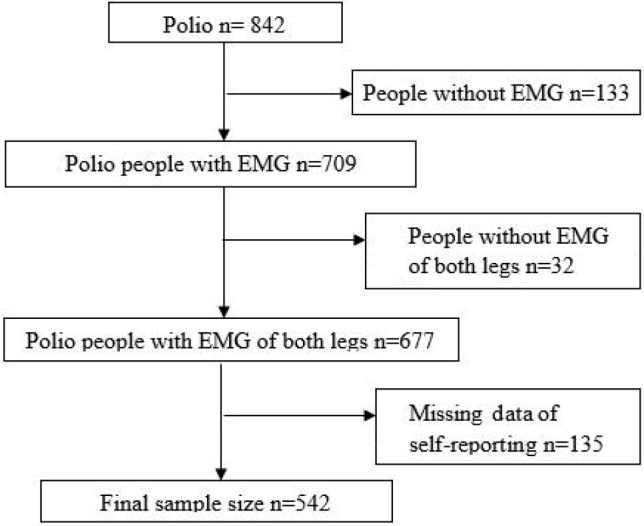
Flowchart of the cohort

Of the 842 people listed at the polio clinic, Sahlgrenska University Hospital, 165 were excluded due to lack of EMG data for the lower limbs. Another 135 people were excluded due to a missing self-reported questionnaire, leaving 542 people available for the study (Fig. 1). Among these 542 people, 57% were female (*n* = 312), the mean age at first visit was 58 years, and the mean age at onset of symptoms deterioration was 50 years. The majority were born in Europe (*n* = 446) (Table I). Three hundred and eight patients (56%) were classified as being aware of impairments, meaning they specified a weakness that was confirmed as polio by EMG, and 234 patients (43%) were classified as having impairments that they were unaware of, meaning they did not report a weakness in that leg. There were no comorbidities (such as stroke or disc hernia) that could result in objective or perceived weaknesses.

**Table I:** Descriptive characteristics of the study sample

Factor	Aware group	Unaware group
Total, *n* (%)	308 (56)	234 (43)
Age at time of visit	59 (14), 18/88	57 (14), 16/84
Time since onset of polio	51 (12), 16/81	50 (12), 12/79
Age at onset of polio	7 (8), 0/37	6 (7), 0/36
Sex, *n* (%)		
Female	179 (58)	133 (57)
Male	129 (42)	101 (43)
Country of birth, *n* (%)		
From Europe, *n* (%)	263 (85)	183 (78)
Outside Europe, *n* (%)	45 (15)	51 (22)

All ages in years, mean (SD), min/max.

**Fig. 2:**
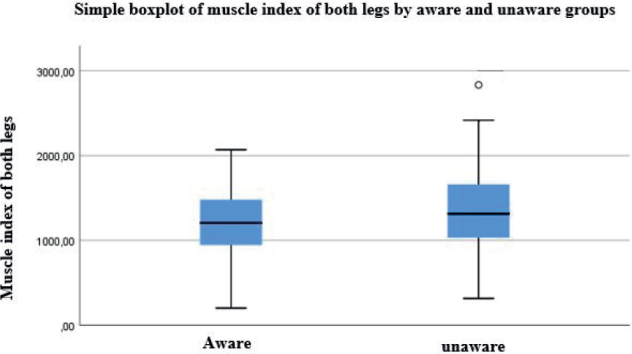
Boxplots of the muscle index (measure of strength) of the aware group (left) and the unaware group. The unaware group is significantly stronger.

There was a significant difference on the30-m walk test (*p* = 0.011) (Table II). The average maximal speed for the aware group (*n* = 250) was (1.18 m/s), with 40% of people scoring significantly slower than control values. In the unaware group (*n* = 201), the maximal walking speed was (1.29 m/s), with 35% of people walking significantly slower than control values. A significant difference in muscle strength index between the aware and unaware groups was noted (*p* = 0.018), with the aware group being weaker (Fig. 2). Wheelchair dependence was 21% (*n* = 55) in the aware group, and 6% (*n* = 14) in the unaware group (χ² = 16, df = 1, *p* < 0.001). EMG could identify signs of chronic muscle denervation in the lower limb of people who reported no previous engagement with polio in that limb, comprising 44% of individuals (*n* = 239) (Table III).

**Table II:** Difference in 30-mwalk test at first visit and muscle index for aware and unaware groups

Factor	Aware group	Unaware group	*p*-value
30-m walk test mean (SD)	0.6110 (0.22620)	0.6625 (0.19593)	0.011
Muscle index, mean (SD)	1221 (369)	1368 (474)	0.018

Missing values: 30-m walk test, *n* = 83 (15%), muscle index, *n* = 341 (62%).

SD: standard deviation.

**Table III:** Ability of subjective experience to detect post-polio muscle weakness at the current stage where polio was revealed by electromyography results

Factor	Right leg, *n* (%)	Left leg, *n* (%)
True positive	370 (68.3%)	378 (69.7%)
False positive	13 (2.4%)	14 (2.6%)
True negative	46 (8.5%)	24 (4.4%)
False negative	113 (20.8%)	126 (23.2%)

## DISCUSSION

The data suggest that EMG to a large extent identifies the presence of chronic muscle denervation associated with muscle weakness in people with late effects of polio with uncertain degree of residuals of the infection. These finding stresses the importance of an EMG examinations in people with a history of polio, regardless of whether or not they present a history of muscle weakness in a limb.

Our results confirm the results of Luciano et al. (11) in a study of 10 individuals with polio. They performed macro EMG on the clinically unaffected gastrocnemius and found increased median amplitude and area of the macro-motor unit potentials, with changes in 8 out of 10 muscles tested. This indicates prior polio engagement in muscles thought to be normal.

Regarding the 30-m walk test, the data were compared with normalized values for sex and age. The reference values were based on a population living in Sweden.

The comparison between the 2 groups showed that the unaware group was faster than the aware group, suggesting a possible risk for overuse of muscles (overuse is when a muscle is repeatedly stressed and never gets a chance to rest).

The difference was even more evident in the use of mobility aids, where the unaware group is significantly less likely to use manual or electric wheelchair. Considering using of mobility aids, we tried to simplify the understanding of dependence on mobility aids by dividing all types of aids into 2 categories. Although individuals may be unaware of their weakness, they still might use advanced mobility aids due to pronounced weakness in the other leg.

The muscle strength index summarized the 8 muscles’ strength measurement to simplify the analysis. Values from muscle groups around the hip were not included in the study due to feasibility issues in measurement. The strength index in the unaware group with late effects of polio was significantly higher than that in the aware group, indicating stronger muscles in unaware people. This implies that overuse of muscles can be more common in individuals unaware of their condition in terms of late effects of polio compared with the aware group.

Unawareness of impairment is due to a compensatory effect, adaptative mechanism, and lack of reference. This may have unintended consequences that can affect muscular functionality. The clinical implication of this study is to increase patients’ awareness to avoid or decrease the overuse of muscles during daily activity based on the results from the EMG. This valuable clinical information can assist the rehabilitation team in providing successful rehabilitation and developing appropriate plans that fit people’s situation and achieve rehabilitation goals. It is important to note that, when we refer to weakness, it means subjective experience during the acute phase and not at the time of first visit.

One strength of this study is its reliance on clinical material registry data from a tax-funded hospital, increasing the generalizability of the data. A limitation of this study was the some missing subjective and objective data for different reasons such as people refusing to participate, being too weak to perform testes, had to no time to participate, EMG not performed, missing information in the questionnaire, or uncertainty regarding the affected body parts during the acute phase. Another weakness is that CT/MR of the lower extremity is not part of the standard clinical examination, which is an excellent alternative to EMG to detect polio involvement by fatty infiltration (16).

In conclusion, this cross-sectional study demonstrates the effectiveness of EMG in identifying chronic muscle denervation associated with weaknesses in people with late effects of polio who perceived themselves as healthy. The take-home message from this study is to perform EMG on people with polio in muscle groups of both legs, even if they exhibit clinical weakness on only one side.
